# Prevalence of Tick-Borne Pathogens in *Ixodes ricinus* and *Dermacentor reticulatus* Ticks from Different Geographical Locations in Belarus

**DOI:** 10.1371/journal.pone.0054476

**Published:** 2013-01-22

**Authors:** Anna L. Reye, Valentina Stegniy, Nina P. Mishaeva, Sviataslau Velhin, Judith M. Hübschen, George Ignatyev, Claude P. Muller

**Affiliations:** 1 Institute of Immunology, Centre de Recherche Public de la Santé / National Public Health Laboratory, Luxembourg, Luxembourg; 2 Clinical and Experimental Laboratory for Chronic Neuroinfections, Republican Research and Practical Centre for Epidemiology and Microbiology, Minsk, Belarus; Kansas State University, United States of Ameica

## Abstract

Worldwide, ticks are important vectors of human and animal pathogens. Besides Lyme Borreliosis, a variety of other bacterial and protozoal tick-borne infections are of medical interest in Europe. In this study, 553 questing and feeding *Ixodes ricinus* (n = 327) and *Dermacentor reticulatus* ticks (n = 226) were analysed by PCR for *Borrelia*, *Rickettsia*, *Anaplasma*, *Coxiella*, *Francisella* and *Babesia* species. Overall, the pathogen prevalence in ticks was 30.6% for *I. ricinus* and 45.6% for *D. reticulatus*. The majority of infections were caused by members of the spotted-fever group rickettsiae (24.4%), 9.4% of ticks were positive for *Borrelia burgdorferi* sensu lato, with *Borrelia afzelii* being the most frequently detected species (40.4%). Pathogens with low prevalence rates in ticks were *Anaplasma phagocytophilum* (2.2%), *Coxiella burnetii* (0.9%), *Francisella tularensis* subspecies (0.7%), *Bartonella henselae* (0.7%), *Babesia microti* (0.5%) and *Babesia venatorum* (0.4%). On a regional level, hotspots of pathogens were identified for *A. phagocytophilum* (12.5–17.2%), *F. tularensis* ssp. (5.5%) and *C. burnetii* (9.1%), suggesting established zoonotic cycles of these pathogens at least at these sites. Our survey revealed a high burden of tick-borne pathogens in questing and feeding *I. ricinus* and *D. reticulatus* ticks collected in different regions in Belarus, indicating a potential risk for humans and animals. Identified hotspots of infected ticks should be included in future surveillance studies, especially when *F. tularensis* ssp. and *C. burnetii* are involved.

## Introduction

Throughout the world, ticks are important vectors of human and animal pathogens. In Eastern Europe, Lyme Borreliosis (LB) is a major public health threat with annual incidence rates ranging from 4.8 (Poland) to 35 (Lithuania) cases per 100.000 population [1]. In Belarus, 1.0 to 9.1 cases per 100.000 have been reported over a ten year period [2]. Although early manifestations of the disease usually can be diagnosed and treated successfully, late manifestations such as Lyme arthritis, Acrodermatitis chronica atrophicans and neuroborreliosis can be severe. The causative agents are members of the *Borrelia burgdorferi* sensu lato (s.l.) complex, of which at least *Borrelia burgdorferi* sensu stricto (s.s.), *B. afzelii* and *B. garinii* are known to be human pathogens, whereas pathogenicity has not been confirmed for *B. spielmanii*, *B. lusitaniae* and *B. valaisiana*. In the neighbouring countries of Belarus, the prevalence of *Borrelia* species in questing *Ixodes* ticks ranges from 6.2% in Poland, 11% in Lithuania to 40.7% in Russia and 18–51% in Latvia [3]–[6]. No such prevalence data are available for Belarus, but the three human pathogenic *Borrelia* species have been isolated from ticks before [7].

Six other tick-borne bacterial and protozoal pathogen genera are of medical interest in Europe. *Rickettsia* species of the spotted fever group (SFG) can cause rickettsioses in humans, which are mainly characterized by feverish infections. Little is known about the incidence of spotted fever rickettsioses in Eastern Europe, but surveillance studies revealed that *Rickettsia* prevalence in ticks ranges from about 3% in Poland and 6% in the Ukraine and Slovakia to 15% in Russia [8]–[11].

*Anaplasma phagocytophilum* is another rickettsial agent pathogenic for humans, causing human granulocytic anaplasmosis. Although the incidence of anaplasmosis in Europe is not well documented, clinical cases seem to be rare. In ticks, a prevalence of 2.9% in Lithuania, 2.9–8.7% in Poland, 3.6% in the Ukraine and 5.0% in Russia have been observed [3], [4], [10], [12], [13].

*Coxiella burnetii* is the causative agent of Q fever in humans, but it also affects domestic and wild ruminants, leading to increased rates of abortion. Although ticks are competent vectors of this pathogen, so far Q fever outbreaks have been linked to exposure to infected animals and their products. Despite only limited studies the prevalence of *C. burnetii* in European ticks, does not seem to exceed 2.6% [9], [14]–[16].

Another tick-borne pathogen that can also be transmitted by aerosol is *Francisella tularensis*, causing tularaemia in humans. Prevalence rates in ticks are at least in France and Germany below 1.6% [17], [18] and like Q fever, tularaemia outbreaks are usually linked to the exposure to contaminated biological matter (faeces, blood, milk, etc.) rather than tick bites.

*Bartonella* species can be transmitted to humans by various arthropods including ticks and cause diseases like trench fever and cat scratch disease. The prevalence of these bacteria in ticks can vary as much as from 3.7% in Poland to 40% in Russia and France [19]–[21].

Besides these bacterial agents, also human pathogenic protozoans can be transmitted by tick bites. At least two *Babesia* species, namely *B. microti* and *B. divergens*, are known to cause babesiosis in humans, whereas for *B. venatorum* (previously *Babesia* sp. EU1) human pathogenicity is suspected. In Poland, 3.5% to 5.4% of ticks were found to be infected with *B. microti* [22], [23], whereas 1.6% of ticks from Russia were infected with *B. venatorum* [8].

The wide distribution of bacterial and protozoal agents of human pathogenicity and the extreme range of their prevalence in ticks indicate the need for comprehensive studies on tick-borne pathogens in particular in Eastern Europe. So far, comparatively few studies on the prevalence of tick-borne pathogens in ticks from Belarus have been published in peer-reviewed journals [24], [25]. However, official data on LB incidence and limited information on *Borrelia burgdorferi* s.l. prevalence in ticks exist [2]. In this study, we investigated the prevalence of seven tick-borne pathogen genera of medical interest in questing and feeding *Ixodes ricinus* and *Dermacentor reticulatus* ticks in Belarus.

## Materials and Methods

Belarus is a landlocked country in North-Eastern Europe with a population of 9.4 million, 70% of which reside in urban areas. Its terrain is generally flat, with an average elevation of 160m above sea level and about 40% of the country is covered by forest. The most common tick species in Belarus are *Ixodes ricinus* and *Dermacentor reticulatus* [2].

**Figure 1:**
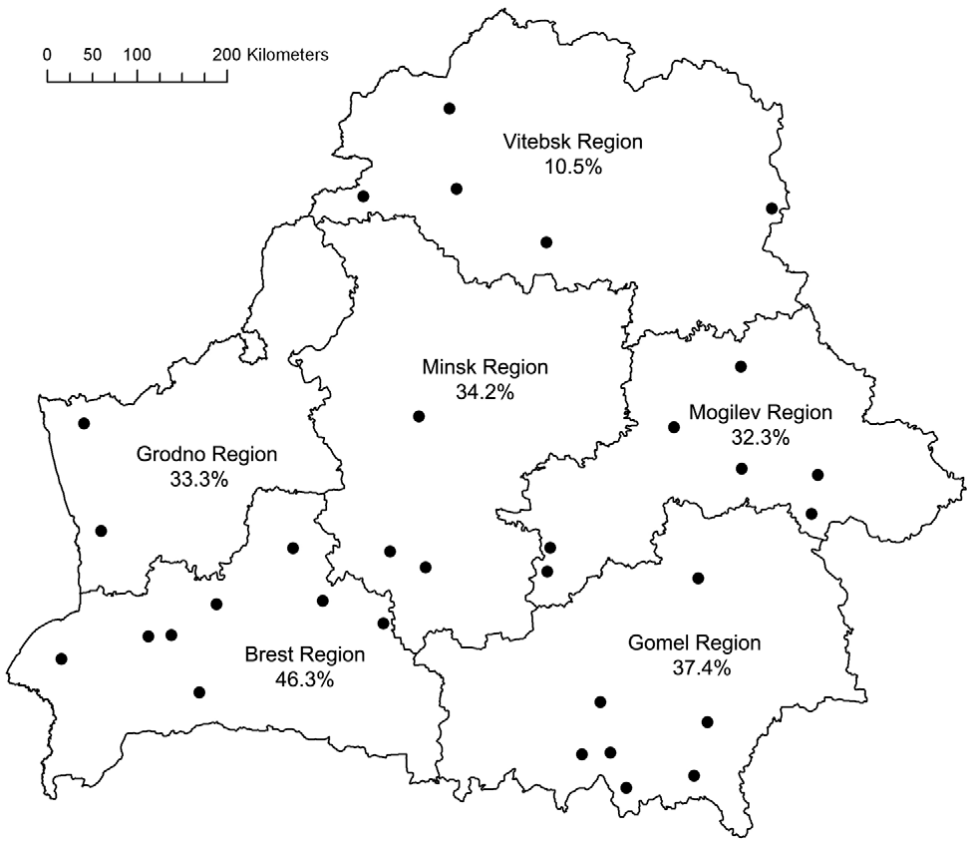
Administrative regions of Belarus showing the 32 collection sites and the total pathogen prevalence in ticks collected from each region.

In April and May 2009, ticks were collected from the vegetation and from cows at 32 collection sites in the administrative regions of Brest (n = 8), Gomel (n = 7), Grodno (n = 2), Minsk (n = 3), Mogilev (n = 7) and Vitebsk (n = 5) (Figure 1). One tick was removed from a dog in Minsk region. Verbal authorisation for collection of feeding ticks was obtained from farmers and animal owners. Collection of questing ticks was performed with the cloth-dragging method; feeding ticks were removed with forceps. Ticks were identified to species level using standard morphological identification keys [26] and stored in 70% ethanol at 4°C. Ticks were washed three times in 1x phosphate buffered saline, rinsed with distilled water and dried on sterile filter paper prior to DNA extraction. Disruption and homogenization was performed in lysis buffer of the InviMag Tissue DNA Mini kit (Invitek, Berlin, Germany) using the TissueLyser II (Qiagen, Venlo, Netherlands) and 5 mm stainless steel beads. The KingFisher 96 Magnetic Particle Processor (Thermo Scientific, Waltham, Massachusetts, USA) was used for the extraction of DNA according to the manufacturers’ instructions. Detection PCRs for *A. phagocytophilum*, *Bartonella* sp., *Babesia* sp., *Borrelia* sp., *C. burnetii*, *Francisella* sp. and *Rickettsia* sp. were carried out using 5µl of raw DNA extract and 1µl of first round products. In addition to the 17kDa PCR used for detection of *Rickettsia* species, a second PCR assay based on the ompA gene was performed for better species discrimination. Primer sequences and PCR conditions are specified in Table 1. In each PCR run, cloned fragments of target genes of the respective pathogens were included as specific positive controls while distilled water was used as negative control. Spike experiments with culture extract from *Borrelia garinii* showed a similar PCR sensitivity in DNA extracts derived from feeding and questing ticks.

**Table 1:** Primers and PCR conditions used for the detection of the eight different pathogen groups.

Pathogen	Primer name	Primer orientation	Target gene	5′-3′ Sequence	Ref.	Primer C	MgCl_2_ C	Annealing step	Elongation step	Fragment length
*Anaplasma phagocytophilum*	EL(569)F	forward	groEL gene	ATGGTATGCAGTTTGATCGC	[46]	0.8 µM	2 mM	61°C 30 s	72°C 45 s	624 bp
	EL(1193)R	reverse	groEL gene	TCTACTCTGTCTTTGCGTTC	[46]					
	EL(569)F	forward	groEL gene	ATGGTATGCAGTTTGATCGC	[46]	0.8 µM	2 mM	56°C 30 s	72°C 45 s	573 bp
	EL(1142)R	reverse	groEL gene	TTGAGTACAGCAACACCACCGGAA	[46]					
*Babesia* sp.	BJ1	forward	18S rRNA	GTCTTGTAATTGGAATGATGG	[47]	0.8 µM	3 mM	61°C 30 s	70°C 60 s	476–520 bp
	BN2	reverse	18S rRNA	TAGTTTATGGTTAGGACTACG	[47]					
*Bartonella* sp.	BH1	forward	groEL gene	GAAGAAACAACTTCTGACTATG	[21]	0.8 µM	1.5 mM	60°C 30 s	72°C 30 s	440 bp
	BH4	reverse	groEL gene	CGCACAACCTTCACAGGATC	[21]					
	HSPps1	forward	groEL gene	CAGAAGTTGAAGTGAAAGAAAA	[48]	0.4 µM	1.5 mM	56°C 30 s	72°C 30 s	350 bp
	BH4	reverse	groEL gene	CGCACAACCTTCACAGGATC	[21]					
*Borrelia burgdorferi* s.l.	Outer1	forward	flaB gene	AARGAATTGGCAGTTCAATC	[49]	0.8 µM	2 mM	59°C 30 s	72°C 30 s	497 bp
	Outer2	reverse	flaB gene	GCATTTTCWATTTTAGCAAGTGATG	[49]					
	Inner1	forward	flaB gene	ACATATTCAGATGCAGACAGAGGTTCTA	[49]	0.8 µM	2 mM	59°C 30 s	72°C 30 s	389 bp
	Inner2	reverse	flaB gene	GAAGGTGCTGTAGCAGGTGCTGGCTGT	[49]					
*Coxiella* sp.	Q5	forward	htpB gene	GCGGGTGATGGTACCACAACA	[50]	0.4 µM	1.5 mM	58°C 30 s	72°C 30 s	501 bp
	Q3	reverse	htpB gene	GGCAATCACCAATAAGGGCCG	[50]					
	Q6	forward	htpB gene	TTGCTGGAATGAACCCCA	[50]	0.4 µM	1.5 mM	56°C 30 s	72°C 30 s	325 bp
	Q4	reverse	htpB gene	TCAAGCTCCGCACTCATG	[50]					
*Francisella tularensis* ssp.	Fr153F0.1	forward	16S rRNA	GCCCATTTGAGGGGGATACC	[51]	0.4 µM	2 mM	60°C 30 s	72°C 60 s	1170 bp
	Fr1281R0.1	reverse	16S rRNA	GGACTAAGAGTACCTTTTTGAGT	[51]					
*Rickettsia* sp.	Rr17k.1p	forward	17-kDa	TTTACAAAATTCTAAAAACCAT	[52]	0.8 µM	2 mM	55°C 30 s	72°C 45 s	539 bp
	Rr17k.539n	reverse	17-kDa	TCAATTCACAACTTGCCATT	[52]					
	Rr17k.90p	forward	17-kDa	GCTCTTGCAACTTCTATGTT	[52]	0.8 µM	2 mM	54°C 30 s	72°C 45 s	450 bp
	Rr17k.539n	reverse	17-kDa	TCAATTCACAACTTGCCATT	[52]					
	Rr 190.70p	forward	ompA	ATGGCGAATATTTCTCCAAAA	[53]	0.4 µM	2 mM	56°C 30 s	72°C 45 s	630 bp
	Rr 190.701	reverse	ompA	GTTCCGTTAATGGCAGCATCT	[54]					
	Rr 190.70p	forward	ompA	ATGGCGAATATTTCTCCAAAA	[53]	0.4 µM	2 mM	58°C 30 s	72°C 45 s	532 bp
	Rr190.602n	reverse	ompA	AGTGCAGCATTCGCTCCCCCT	[53]					

C, concentration; bp, base pairs

PCR protocol: 94°C for 3 min; 40 cycles of 94°C for 30 s, specific annealing conditions and 72°C for specific elongation time; subsequent incubation at 72°C for 10 min.

Sequencing and phylogenetic analyses were carried out as described before [14] and sequences are available at NCBI under accession numbers JQ711209-JQ711262 and JX648098-JX648109. Statistical analyses were performed with Fisher’s exact test (whenever a cell value of the contingency table was equal to or below 5) or Pearson’s goodness of fit chi-square (GFX) test; *P* values < 0.05 are given in the text.

## Results

### Tick collection

In total, 553 ticks belonging either to the species *Ixodes ricinus* (59.1%; 327/553) or *Dermacentor reticulatus* (40.9%; 226/553), were collected from the vegetation (81.9%; 453/553), cattle (17.9%; 99/553) and a dog (0.2%; 1/553). The majority of ticks collected from the vegetation were *I. ricinus* (63.8%; 289/453), whereas from cattle mostly *D. reticulatus* was removed (61.6%; 61/99) (Table 2). Most ticks were collected in Gomel region (53.7%; 297/553), followed by Brest (14.8%; 82/553) and Minsk region (14.3%; 79/553). Only few ticks were collected in Grodno (8.1%; 45/553), Mogilev (5.6%; 31/553) and Vitebsk region (3.4%; 19/553) (Table 2). Adults were the predominant instars (99.6%; 551/553) comprising of 59.5% females (329/553) and 40.1% males (222/553). Only two nymphal *I. ricinus* (0.4%) were collected from the vegetation (Table 2). In the regions Brest, Gomel and Minsk, both tick species were equally prevalent (50 ±5%), whereas *I. ricinus* was predominant in Mogilev, Grodno and Vitebsk (67.7–97.8%) (Table 2). The overall prevalence of infected questing ticks from Belarus (counting mixed infected ticks only once) was 37.7% (171/453). Questing *I. ricinus* displayed an infection prevalence of 32.9% (95/289), which was significantly lower than for *D. reticulatus* (46.3%; 76/164; p<0.01) (Table 3). The overall prevalence of infections in feeding ticks was 32% (32/100); significantly fewer feeding *I. ricinus* ticks were positive in the pathogen detection PCRs (13.2%; 5/38) than feeding *D. reticulatus* ticks (43.5%; 27/62; p<0.001). On a regional level, there were considerable differences in the total prevalence of infected ticks, ranging from 10.5% in Vitebsk to 46.3% in Brest (see Figure 1).

**Table 2:** Numbers of questing and feeding ticks collected in different regions of Belarus.

Ticks	Source		Brest	Gomel	Grodno	Minsk	Mogilev	Vitebsk	Total
**I. ricinus**			40	168	44	37	21	17	327
	Vegetation	T	40	163	44	37	–	5	289
	Vegetation	F	18	82	30	16	–	1	147
	Vegetation	M	22	81	14	19	–	4	140
	Vegetation	N	–	–	–	2	–	–	2
	Host	T	–	5		–	21	12	38
	Host	F	–	5	–	–	18	12	35
	Host	M	–	–	–	–	3	–	3
	Host	N	–	–	–	–	–	–	–
**D.reticulatus**			42	129	1	42	10	2	226
	Vegetation	T	14	106	1	41	2	–	164
	Vegetation	F	7	66	1	12	1	–	87
	Vegetation	M	7	40	–	29	1	–	77
	Vegetation	N	–	–	–	–	–	–	–
	Host	T	28	23	–	1	8	2	62
	Host	F	27	23	–	1	7	2	60
	Host	M	1	–	–	–	1	–	2
	Host	N	–	–	–	–	–	–	–

I, Ixodes; D, Dermacentor; T, Total; F, Female; M, Male; N, Nymph; -, not found.

**Table 3:** Pathogen prevalence in questing and feeding ticks.

	Total		*I. ricinus*		*D. reticulatus*	
Pathogen species	H (%)	V (%)	H (%)	V (%)	H (%)	V (%)
A. phagocytophilum	–	12 (2.6)	–	12 (4.2)	–	–
Ba. microti	–	3 (0.7)	–	3 (1.0)	–	–
Ba. venatorum	–	2 (0.4)	–	2 (0.7)	–	–
Bt. henselae	–	4 (0.9)	–	3 (1.0)	–	1 (0.6)
B. burgdorferi s.l.	5 (5.0)	47 (10.4)	2 (5.3)	46 (14.1)	3 (4.8)	3 (1.8)
B. afzelii	2 (2.0)	19 (4.2)	2 (5.3)	18 (6.2)	–	1 (0.6)
B. burgdorferi s.s.	2 (2.0)	8 (1.8)	–	6 (2.1)	2 (3.2)	2 (1.2)
B. garinii	–	11 (2.4)	–	11 (3.8)	–	–
B. lusitaniae	–	1 (0.2)	–	1 (0.3)	–	–
B. valaisiana	1 (1.0)	8 (1.8)	1 (2.6)	7 (2.4)	–	1 (0.6)
C. burnetii	–	5 (1.1)	–	5 (1.7)	–	–
F. tularensis	–	4 (0.9)	–	4 (1.4)	–	–
Rickettsia species	28 (28)	107 (23.6)	2 (5.3)	34 (11.7)	26 (41.9)	73 (44.5)
R. helvetica	1 (1.0)	29 (6.4)	–	29 (10.0)	1 (1.6)	–
R. monacensis	–	5 (1.1)	–	5 (1.7)	–	–
R. raoultii	23 (23.0)	37 (8.2)	1 (2.6)	–	22 (35.5)	37 (22.6)
RRG	4 (4.0)	36 (7.9)	1 (2.6)	–	3 (4.8)	36 (22.0)
Total*	32 (32.0)	171 (37.7)	5 (13.2)	95 (32.97)	27 (43.5)	76 (46.3)

I, Ixodes; D, Dermacentor; H, Host; V, Vegetation;-, not found; A, Anaplasma; Ba, Babesia; Bt, Bartonella;

B, Borrelia; s.l., sensu lato; C, Coxiella; F, Francisella; R, Rickettsia; RRG, Rickettsia rickettsii group.

* Note that ticks with mixed infections are only counted once.

### Rickettsiaceae

**Figure 2:**
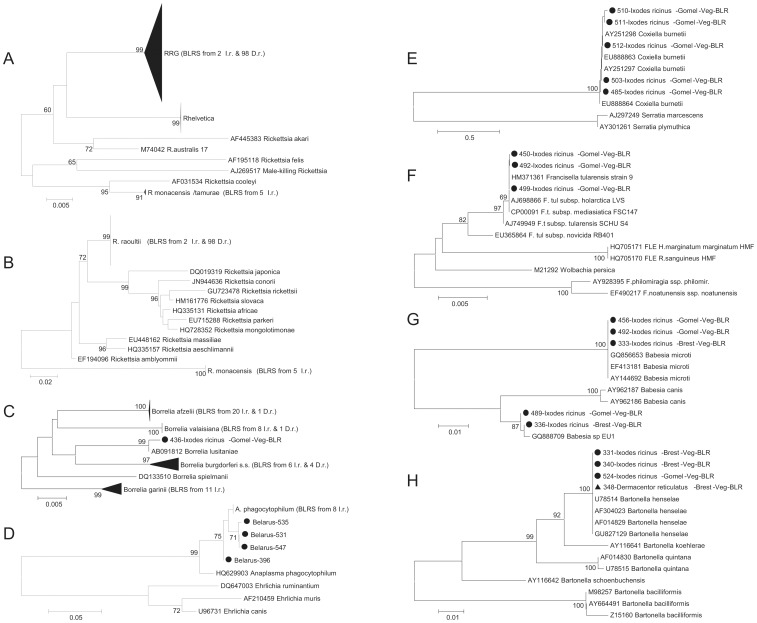
Phylogenetic trees for speciation of pathogens in Belarus. Neighbour-Joining trees based on (A) a 344 nt fragment of the 17-kDa gene of *Rickettsia* species (nt 1194706 –1195039 of CP000766.2), (B) a 395 nt fragment of the ompA gene of *Rickettsia* species (nt 71 –465 of JN400406.1), (C) a 348 nt fragment of the FlaB gene of *Borrelia burgdorferi* s.l. (nt 97–444 of HM345909.1), (D) a 352 nt fragment of the groEL gene of *Anaplasma* species (nt 732–1083 of HQ629903.1), (E) a 319 nt fragment of the htpB gene of *Coxiella burnetii* (nt 320–638 of EU888863.1), (F) a 894 nt fragment of the 16S rRNA gene of *Francisella* species (nt 8–898 of HM371361.1), (G) a 515 nt fragment of the 18S rRNA gene of *Babesia* species (nt 2–516 of GQ856653.1) and (H) a 320 nt fragment of the groEL gene of *Bartonella* species (nt 14–333 of GU827129.1). Sequences from Belarus are indicated by solid circles (*Ixodes ricinus*) or triangles (*Dermacentor reticulatus*) and named with their unique identifier, tick species, geographic location, biological source and WHO country code. Only sequences of the denoted lengths were included in the displayed phylogenies. The number of sequences from Belarus within a compressed cluster and the tick species are given in brackets. BLRS, sequences from Belarus obtained in this study; I.r.  =  *Ixodes ricinus*; D.r.  =  *Dermacentor reticulatus*; RRG, *Rickettsia rickettsii* group; Veg  =  Vegetation, BLR, Belarus. Only bootstrap values above 60 are shown.

Spotted Fever Group (SFG) Rickettsia were detected in 24.4% (135/553) of ticks using the 17kDa PCR assay, of which 22.2% (30/135) were identified as *Rickettsia helvetica* and 3.7% (5/135) clustered with *Rickettsia monacensis*/*Rickettsia tamurae* (Figure 2A; Table 3). The remaining sequences (74.1%; 100/135) clustered with the *Rickettsia* species *R. amblyommii, R. conorii, R. heilongjiangii, R. honei, R. japonica, R. marmionii, R. montana, R. montanensis, R. parkeri, R. peacockii, R. rhipicephali, R. rickettsia, R. sibirica, and R. slovaca,* hereafter referred to as the *Rickettsia rickettsii* group (RRG) (Figure 2A; Table 3). From the latter 105 samples, the discriminatory ompA PCR generated 65 amplicons (61.9%; 65/105), corresponding to *R. monacensis* (5/5) and *Rickettsia raoultii* (60/100) (Table 3). Forty RRG positive samples were negative in the ompA PCR assay.

The *Rickettsia* infection rate was significantly higher (p<0.01) in *D. reticulatus* (43.8%; 99/226) than *I. ricinus* (11.0%; 36/327). In questing *D. reticulatus* ticks, RRG was the most prevalent species (22.0%; 36/164), followed by *R. raoultii* (19.5%; 39/99) (Table 3). Feeding *D. reticulatus* ticks were predominantly infected by *R. raoultii* (32.3%; 20/62), followed by RRG (4.8%; 3/62). The only *D. reticulatus* tick harbouring *R. helvetica* (1.0%; 1/99) was feeding on a dog. In questing *I. ricinus* ticks, the prevalence was 10.0% (29/289) for *R. helvetica* and 1.7% (5/289) for *R. monacensis*; *R. raoultii* and RRG were only detected in feeding *I. ricinus* (5.3%; 2/38) (Table 3). On a regional level, *R. raoultii* displayed highest prevalence rates in ticks collected in the regions of Brest (20.7%; 17/82) and Mogilev (16.1%; 5/31), whereas ticks positive for RRG and *R. helvetica* were predominantly found in Gomel (9.8%; 29/297) and Grodno region (15.6%; 7/45), respectively. All *R. monacensis* infected ticks were collected at the same site in Minsk region.

### *Borrelia* species

*Borrelia burgdorferi* sensu lato was the second most prevalent pathogen and detected in 9.4% (52/553) of all ticks. The predominant species was *B. afzelii* (40.4%; 21/52) followed by *B. garinii* (21.2%; 11/52), *B. burgdorferi* sensu stricto (s.s.) (19.2%; 10/52), *B.valaisiana* (17.3%; 9/52) and *B. lusitaniae* (1.9%; 1/52) (Table 3, Figure 2B). The *Borrelia* prevalence was significantly higher (p<0.05) in *I. ricinus* (14.1%; 46/327) than in *D. reticulatus* (2.7%; 6/226). *I. ricinus* ticks were infected with all *Borrelia* species detected, whereas in *D. reticulatus* ticks only *B. burgdorferi* s.s., *B. afzelii* and *B. valaisiana* were detected (Table 3, Figure 2B). In ticks from the vegetation, the *Borrelia* prevalence (10.4%; 47/453) was twice as high as in those from host animals (5.0%; 5/100) (Table 3). Interestingly, the prevalence of *Borrelia* infected ticks was considerably higher in Brest (15.9%; 13/82) and Grodno (15.6%; 7/45) than in Gomel (7.7%; 23/297; p<0.05) and the remaining regions (5.3%–9.7%; not significant). *Borrelia* species diversity was highest in ticks from Gomel (all 5 species detected) and lowest in Vitebsk region (only *B. garinii*).

### Low prevalent pathogens

The other pathogens were exclusively detected in questing ticks. *Anaplasma phagocytophilum* (2.6%; 12/453), *Coxiella burnetii* (1.1%; 5/453), *Francisella tularensis* ssp. (0.9%; 4/453), *Babesia microti* (0.7%; 3/453) and *Babesia venatorum* (0.4%; 2/453) were only detected in *I. ricinus* ticks, whereas both tick species harboured *Bartonella henselae* (0.9%; 4/453) (Table 3, Figures 2C-G). *A. phagocytophilum* was detected in three regions and its prevalence in ticks was considerably higher in Minsk (6.3%; 8/79) than in Gomel (2.0%; 6/297; p<0.05) and Grodno (2.2%; 1/45; not significant). Ticks from Brest and Gomel region were infected with *B. henselae* and both *Babesia* species, whereas only ticks from Gomel harboured *F. tularensis* ssp. and *C. burnetii*.

### Pathogens in questing and feeding ticks

Overall, the pathogen species composition was significantly more diverse in questing than in feeding ticks (14 vs. 4 species; p<0.05). Questing *I. ricinus* ticks were infected with more pathogen species than questing *D. reticulatus* (13 vs. 5 species; not significant) (Table 4). Interestingly, the pathogen prevalence in *I. ricinus* was significantly lower in feeding than in questing ticks (13.2% vs. 32.9%; p<0.01), whereas it was similar in *D. reticulatus* ticks (42.6% vs. 46.3%; not significant). Mixed infections were detected in 2.5% (14/553) of ticks, the majority of which were formed between members of the two most prevalent pathogen genera *Rickettsia* and *Borrelia* (57.1%; 8/14) (Table 5). Also, mixed infections occurred more often in *I. ricinus* than in *D. reticulatus* ticks (3.4% vs. 1.3%; not significant). Gender specific differences in the pathogen prevalence in ticks could not be evaluated due to the low number of feeding male ticks (n = 5).

**Table 4:** Pathogen diversity in questing I. ricinus and D. reticulatus ticks.

Pathogen species	Prevalence in I.ricinus (%)	Prevalence in D.reticulatus (%)
A. phagocytophilum	12 (4.2)	–
B. afzelii	18 (6.2)	1 (0.6)
B. burgdorferi s.s.	6 (2.1)	2 (1.2)
B. garinii	11 (3.8)	–
B. lusitaniae	1 (0.3)	–
B. valaisiana	7 (2.4)	2 (1.2)
Ba. microti	3 (1.0)	–
Ba. venatorum	2 (0.7)	–
Bt. henselae	3 (1.0)	1 (0.6)
C. burnetii	5 (1.7)	–
F. tularensis ssp.	4 (1.4)	–
R. raoultii	–	37 (22.6)
R. helvetica	29 (10.0)	–
R. monacensis	5 (1.7)	–
RRG	–	36 (22.0)

I., Ixodes; D., Dermacentor; A., Anaplasma; B., Borrelia, Ba., Babesia; Bt., Bartonella; C., Coxiella; F., Francisella; R., Rickettsia; RRG, Rickettsia rickettsii group; s.s., sensu stricto; ssp., subspecies.

**Table 5:** Coinfections in questing and feeding ticks.

Tick species	Sex	Source	Borrelia	Rickettsia	Anaplasma	Babesia	Bartonella	Coxiella	Francisella
D. reticulatus	F	C	burgdorferi s.s.	raoultii	–	–	–	–	–
D. reticulatus	F	V	burgdorferi s.s.	raoultii	–	–	–	–	–
D. reticulatus	F	V	valaisiana	RRG	–	–	–	–	–
I. ricinus	F	V	afzelii	helvetica	–	–	–	–	–
I. ricinus	F	V	afzelii	helvetica	–	–	–	–	–
I. ricinus	F	V	afzelii	helvetica	–	–	–	–	–
I. ricinus	F	V	afzelii	–	–	–	henselae	–	–
I. ricinus	F	V	burgdorferi s.s.	–	phagocytophilum	–	–	–	–
I. ricinus	M	V	garinii	helvetica	–	–	–	–	–
I. ricinus	F	V	garinii	helvetica	–	–	–	–	–
I. ricinus	F	V	valaisiana	–	–	–	–	burnetii	–
I. ricinus	F	V	–	helvetica	–	–	–	burnetii	–
I. ricinus	F	V	–	–	phagocytophilum	–	–	–	tularensis ssp.
I. ricinus	M	V	–	–	–	microti	–	–	tularensis ssp.

D, Dermacentor; I, Ixodes; F, Female; M, Male; C, Cattle; V, Vegetation; RRG, Rickettsia rickettsii group; s.s., sensu stricto; ssp., subspecies.

## Discussion

This is the first comprehensive study on tick-borne bacterial and protozoan pathogens of human and veterinary interest in Eastern Europe. In this study, the ticks collected both from the vegetation and from hosts were mostly adults. This finding is not surprising for ticks removed from cows, as larger mammals serve as the main blood meal host for adult ticks, whereas immature ticks preferably feed on smaller vertebrates. In contrast, on vegetation immature tick stages are normally more abundant than adults due to high interstadial mortality rates [27]. Although cloth dragging has been described as a suitable collection method also for nymphs [28], our results nevertheless suggest that our collection was biased towards adult ticks.

In questing *I. ricinus* ticks, we observed a prevalence of *Rickettsia* species of 11.7%, which was similar to that reported from questing adult *I. ricinus* ticks from Poland (7.8–11.1%) and Slovakia (6.1%) [9], [11], [29]. Also, the prevalence of *Rickettsia* in questing *D. reticulatus* (44.5%) was comparable to reports from Poland (40.7%) [30]. Although not all 100 RRG samples were positive in the discriminatory ompA PCR, all amplified sequences allowed the identification of *R. raoultii* in at least one tick from each RRG positive collection site. *R. raoultii* and RRG were almost exclusively detected in *D. reticulatus,* only two feeding *I. ricinus* ticks were found to be infected by these rickettsiae. This suggests a high adaptation of these bacteria to the former tick species and indeed, high rates of transovarial transmission of 90% to 100% have been reported for *R. raoultii* in *D. reticulatus* and *D. marginatus* ticks [31].

The observed prevalence of *R. raoultii* of 22.6% in questing *D. reticulatus* was comparable to the 22.3% reported from Slovakia [32]. Although *R. raoultii* is endemic in Poland and Russia [31], [33], the prevalence in ticks is not known. *R. raoultii* is a member of the spotted fever group rickettsiae and is suspected to cause tick-borne lymphadenopathy (TIBOLA) in humans [34]. Despite the high prevalence of infected *D. reticulatus* and their implication in human TIBOLA infections, there are no such reports from Belarus available.

The overall prevalence of *Borrelia* infected *I. ricinus* collected from the vegetation was 12.5%, which is in the lower range of the prevalences of 10.1–32.7% reported for questing adult *I. ricinus* from Eastern Europe [35]. For questing *D. reticulatus*, the prevalence of *B. burgdorferi* s.l. (1.8%) was also lower than that reported for questing adult *D. reticulatus* from Russia (3.6%) [21]. On a regional level, the highest *Borrelia* prevalence in ticks of 15.9% was observed in Brest region, which seems to be in line with reports of the highest LB incidence in this region [2]. *B. afzelii* and *B. garinii*, the most prevalent *Borrelia* species in this study, have been previously reported to be predominant in Belarus [25]. However, this is the first report on *B. burgdorferi* s.s. and *B. lusitaniae* prevalence in ticks from Belarus.

Interestingly, the *Borrelia* prevalence was significantly higher in *I. ricinus* ticks collected from the vegetation than in those from cattle. This is surprising, as feeding adult ticks were removed during their third blood meal, whereas questing adults only fed twice. Here, however, the prevalence of feeding and questing adult ticks should be similar as cattle are not considered a competent reservoir host for *B. burgdorferi* s.l. due to the bactericidal activity of bovine serum which results in the complement-mediated lysis of *Borrelia* [36]. Sensitivity testing of our PCR on DNA extracts from feeding and questing ticks spiked with *B. garinii* excluded a difference in detection sensitivity. This suggests that the reduced *Borrelia* prevalence in feeding ticks was not an artifact due to PCR inhibition. Cattle on pastures may serve as an easier blood meal host than wild animals and thus act as a dilution factor for *Borrelia* prevalence in ticks. This inhibitory influence of less competent reservoir hosts on an enzootic cycle has been modeled before [37], [38].

Pathogens of the genera *Anaplasma*, *Babesia*, *Bartonella*, *Coxiella* and *Francisella* were exclusively found in questing ticks from Belarus with low prevalences comparable to reports from questing adult ticks from other Eastern European countries [3], [8], [9], [12], [21], [22], [39], although occasionally also higher prevalences have been reported for these pathogens [3], [21], [40]. The only study from Belarus reports a prevalence of *A. phagocytophilum* in questing *I. ricinus* from Minsk region of 4.2% [24], which is significantly lower than our findings (13.5%; 5/37; p<0.05).

These observed low prevalences in Belarusian ticks are in line with low incidence reports of these diseases in Eastern Europe. However, so far no such incidence data is available for Belarus and there are limited reports from the neighbouring countries, e.g. a single case report of arthropod-borne tularaemia in Poland has been published [41]. Serological studies from Poland and Russia reveal antibody positivities in humans of 5.1 to 11.8% for *Anaplasma phagocytophilum* and 2.6 to 9.0% for *Babesia microti* [42], [43]. Interestingly, a high seroprevalence was reported for *Bartonella henselae* from Poland (23.1 to 37.5%); however, this may be due to other routes of transmission like contact to infected cats rather than to tick bites.

Interestingly, hotspots of infection were discovered at sites in Minsk and Gomel region for *A. phagocytophilum* (12.5–17.2%), *F. tularensis* ssp. (5.5%) and *C. burnetii* (9.1%). The focality of prevalence rates suggests that zoonotic cycles of these pathogens are well established at least at these sites. Since the inhalation of contaminated aerosols (e.g. dried faeces) is an important route of transmission of *F. tularensis* ssp. and *C. burnetii* [44], [45], these sites may represent a significant threat to human health independent of tick exposure. Therefore, studies on the seroprevalence and incidence of these diseases in humans as well as the surveillance of these pathogens not only in ticks but also in reservoir hosts (e.g. members of the Leporidae for *F. tularensis* ssp. and of the Bovidae for *C. burnetii*) at the identified hotspots and neighbouring regions are warranted in order to predict and avoid outbreaks of tularaemia and Q fever.

Gomel region displayed the highest pathogen diversity in the country and is also the region with the largest numbers of ticks, suggesting that it may best reflect the infection status of ticks in Belarus at least for the low prevalent pathogens *Anaplasma, Bartonella, Babesia*, *Coxiella* and *Francisella*. Nevertheless, the prevalence of *Borrelia* infected ticks in this region seemed to be lower than in the western regions Grodno and Brest, even when only *I. ricinus,* a competent vector of *Borrelia* species, is considered. These differences may be due to habitat differences rather than the geographic location.

We found that pathogen diversity was higher in questing *I. ricinus* than in questing *D. reticulatus* ticks, suggesting that the latter species serves as reservoir for fewer pathogens than *I. ricinus*, at least in Belarus. Our survey revealed a high burden of tick-borne pathogens in questing and feeding *I. ricinus* and *D. reticulatus* ticks collected in different regions in Belarus, indicating a potential risk for humans and animals. The pathogenic potential of RRG and the role of *D. reticulatus* as its arthropod vector require further attention. Identified hotspots of infected ticks, especially when *F. tularensis* ssp. and *C. burnetii* are involved, should be included in future surveillance studies. In addition, the impact of the highly prevalent *R. raoultii* and other human pathogens on human health should be assessed in clinical and serological studies.

## References

[1] Lindgren E, Jaenson TGT (2006) Lyme borreliosis in Europe: influences of climate and climate change, epidemiology, ecology and adaptation measures. WHO Regional Office for Europe.

[2] Karaban I, Vedenkov A, Yashkova S, Sebut N (2009) Epidemiology of tick-borne encephalitis and Lyme disease in the Republic of Belarus, 1998-2007. EpiNorth 10: 48–57.

[3] Zygner W, Jaros S, Wedrychowicz H (2008) Prevalence of Babesia canis, Borrelia afzelii, and Anaplasma phagocytophilum infection in hard ticks removed from dogs in Warsaw (central Poland). Vet Parasitol 153: 139–142. 18328630 10.1016/j.vetpar.2008.01.036

[4] Masuzawa T, Kharitonenkov IG, Okamoto Y, Fukui T, Ohashi N (2008) Prevalence of Anaplasma phagocytophilum and its coinfection with Borrelia afzelii in Ixodes ricinus and Ixodes persulcatus ticks inhabiting Tver Province (Russia) - a sympatric region for both tick species. J Med Microbiol 57: 986–991. 18628500 10.1099/jmm.0.47721-0

[5] Bormane A, Lucenko I, Duks A, Mavtchoutko V, Ranka R, et al (2004) Vectors of tick-borne diseases and epidemiological situation in Latvia in 1993-2002. Int J Med Microbiol 293 Suppl 3736–47. 15146983 10.1016/s1433-1128(04)80007-x

[6] Motiejunas L, Bunikis J, Barbour AG, Sadziene A (1994) Lyme borreliosis in Lithuania. Scand J Infect Dis 26: 149–155. 8036470 10.3109/00365549409011778

[7] Trofimov NM, Scheslenok EP, Korenberg EI, Gorelova NB, Postic D, et al.. (1998) [The genotyping of strains of Borrelia burgdorferi sensu lato isolated in Byelarus from Ixodes ricinus ticks]. Med Parazitol (Mosk): 21-22. 10050547

[8] Movila A, Reye AL, Dubinina HV, Tolstenkov OO, Toderas I, et al.. (2010) Detection of Babesia Sp. EU1 and Members of Spotted Fever Group Rickettsiae in Ticks Collected from Migratory Birds at Curonian Spit, North-Western Russia. Vector Borne Zoonotic Dis. 10.1089/vbz.2010.0043 20553110

[9] Smetanova K, Schwarzova K, Kocianova E (2006) Detection of Anaplasma phagocytophilum, Coxiella burnetii, Rickettsia spp., and Borrelia burgdorferi s. l. in Ticks, and wild-living animals in western and middle Slovakia. Ann N Y Acad Sci 1078: 312–315. 17114728 10.1196/annals.1374.058

[10] Movila A, Rolain JM, Podavalenko A, Toderas I, Tkachenco L, et al (2009) Detection of spotted fever group rickettsiae and family Anaplasmataceae in Ixodes ricinus ticks from Republic of Moldova and Eastern Ukraine. Clin Microbiol Infect 15 Suppl 232–33. 10.1111/j.1469-0691.2008.02152.x 19416287

[11] Stanczak J (2006) The occurrence of Spotted Fever Group (SFG) Rickettsiae in Ixodes ricinus ticks (Acari: Ixodidae) in northern Poland. Ann N Y Acad Sci 1078: 512–514. 17114767 10.1196/annals.1374.100

[12] Radzijevskaja J, Paulauskas A, Rosef O (2008) Prevalence of Anaplasma phagocytophilum and Babesia divergens in Ixodes ricinus ticks from Lithuania and Norway. Int J Med Microbiol 298: 218–221.

[13] Grzeszczuk A, Stanczak J, Kubica-Biernat B, Racewicz M, Kruminis-Lozowska W, et al (2004) Human anaplasmosis in north-eastern Poland: seroprevalence in humans and prevalence in Ixodes ricinus ticks. Ann Agric Environ Med 11: 99–103. 15236505

[14] Reye AL, Hübschen JM, Sausy A, Muller CP (2010) Prevalence and seasonality of tick-borne pathogens in questing Ixodes ricinus ticks from Luxembourg. Appl Environ Microbiol 76: 2923–2931. 20228110 10.1128/AEM.03061-09 PMC2863427

[15] Spitalska E, Kocianova E (2003) Detection of Coxiella burnetii in ticks collected in Slovakia and Hungary. Eur J Epidemiol 18: 263–266. 12800953 10.1023/A:1023330222657

[16] Sprong H, Tijsse-Klasen E, Langelaar M, De Bruin A, Fonville M, et al.. (2011) Prevalence of Coxiella Burnetii in Ticks After a Large Outbreak of Q Fever. Zoonoses Public Health. 10.1111/j.1863-2378.2011.01421.x 21824373

[17] Reis C, Cote M, Paul RE, Bonnet S (2010) Questing ticks in suburban forest are infected by at least six tick-borne pathogens. Vector Borne Zoonotic Dis 11: 907–916. 21158500 10.1089/vbz.2010.0103

[18] Franke J, Fritzsch J, Tomaso H, Straube E, Dorn W, et al (2010) Coexistence of pathogens in host-seeking and feeding ticks within a single natural habitat in Central Germany. Appl Environ Microbiol 76: 6829–6836. 20729315 10.1128/AEM.01630-10 PMC2953012

[19] Podsiadly E, Karbowiak G, Tylewska-Wierzbanowska S (2009) Presence of Bartonella spp. in Ixodidae ticks. Clin Microbiol Infect 15 Suppl 2120–121. 10.1111/j.1469-0691.2008.02196.x 19416286

[20] Dietrich F, Schmidgen T, Maggi RG, Richter D, Matuschka FR, et al (2010) Prevalence of Bartonella henselae and Borrelia burgdorferi sensu lato DNA in ixodes ricinus ticks in Europe. Appl Environ Microbiol 76: 1395–1398. 20061459 10.1128/AEM.02788-09 PMC2832386

[21] Rar VA, Fomenko NV, Dobrotvorsky AK, Livanova NN, Rudakova SA, et al (2005) Tickborne pathogen detection, Western Siberia, Russia. Emerg Infect Dis 11: 1708–1715. 16318722 10.3201/eid1111.041195 PMC3367347

[22] Wojcik-Fatla A, Cisak E, Chmielewska-Badora J, Zwolinski J, Buczek A, et al (2006) Prevalence of Babesia microti in Ixodes ricinus ticks from Lublin region (eastern Poland). Ann Agric Environ Med 13: 319–322. 17196008

[23] Wojcik-Fatla A, Szymanska J, Wdowiak L, Buczek A, Dutkiewicz J (2009) Coincidence of three pathogens (Borrelia burgdorferi sensu lato, Anaplasma phagocytophilum and Babesia microti) in Ixodes ricinus ticks in the Lublin macroregion. Ann Agric Environ Med 16: 151–158. 19630205

[24] Katargina O, Geller J, Alekseev A, Dubinina H, Efremova G, et al (2012) Identification of Anaplasma phagocytophilum in tick populations in Estonia, the European part of Russia and Belarus. Clin Microbiol Infect 18: 40–46. 10.1111/j.1469-0691.2010.03457.x 21199155

[25] Postic D, Korenberg E, Gorelova N, Kovalevski YV, Bellenger E, et al (1997) Borrelia burgdorferi sensu lato in Russia and neighbouring countries: high incidence of mixed isolates. Res Microbiol 148: 691–702. 9765854 10.1016/S0923-2508(99)80068-0

[26] Estrada-Peña A, Bouattour A, Camicas J-L, Walker AR (2004) Ticks of Domestic Animals in the Mediterranean Region. A Guide to Identification of Species.

[27] Randolph SE (1998) Ticks are not Insects: Consequences of Contrasting Vector Biology for Transmission Potential. Parasitol Today 14: 186–192. 17040748 10.1016/s0169-4758(98)01224-1

[28] Dobson ADM, Taylor JL, Randolph SE (2011) Tick (Ixodes ricinus) abundance and seasonality at recreational sites in the UK: Hazards in relation to fine-scale habitat types revealed by complementary sampling methods. TTBDIS 2: 67–74. 10.1016/j.ttbdis.2011.03.002 21771540

[29] Stanczak J, Racewicz M, Michalik J, Buczek A (2008) Distribution of Rickettsia helvetica in Ixodes ricinus tick populations in Poland. Int J Med Microbiol 298: 231–234.17765657

[30] Stanczak J (2006) Detection of spotted fever group (SFG) rickettsiae in Dermacentor reticulatus (Acari: Ixodidae) in Poland. Int J Med Microbiol 296 Suppl 40144–148. 16524778 10.1016/j.ijmm.2006.01.014

[31] Samoylenko I, Shpynov S, Raoult D, Rudakov N, Fournier PE (2009) Evaluation of Dermacentor species naturally infected with Rickettsia raoultii. Clin Microbiol Infect 15 Suppl 2305–306. 19438650 10.1111/j.1469-0691.2008.02249.x

[32] Spitalska E, Stefanidesova K, Kocianova E, Boldis V (2012) Rickettsia slovaca and Rickettsia raoultii in Dermacentor marginatus and Dermacentor reticulatus ticks from Slovak Republic. Exp Appl Acarol 57: 189–197. 22392435 10.1007/s10493-012-9539-8

[33] Matsumoto K, Grzeszczuk A, Brouqui P, Raoult D (2009) Rickettsia raoultii and Anaplasma phagocytophilum in Dermacentor reticulatus ticks collected from Bialowieza Primeval Forest European bison (Bison bonasus bonasus), Poland. Clin Microbiol Infect 15 Suppl 2286–287. 10.1111/j.1469-0691.2008.02238.x 19548992

[34] Rieg S, Schmoldt S, Theilacker C, de With K, Wolfel S, et al (2011) Tick-borne lymphadenopathy (TIBOLA) acquired in Southwestern Germany. BMC Infect Dis 11: 167. 21663601 10.1186/1471-2334-11-167 PMC3128054

[35] Rauter C, Hartung T (2005) Prevalence of Borrelia burgdorferi sensu lato genospecies in Ixodes ricinus ticks in Europe: a metaanalysis. Appl Environ Microbiol 71: 7203–7216. 16269760 10.1128/AEM.71.11.7203-7216.2005 PMC1287732

[36] Kurtenbach K, Sewell HS, Ogden NH, Randolph SE, Nuttall PA (1998) Serum complement sensitivity as a key factor in Lyme disease ecology. Infect Immun 66: 1248–1251. 9488421 10.1128/iai.66.3.1248-1251.1998 PMC108041

[37] Norman R, Bowers RG, Begon M, Hudson PJ (1999) Persistence of tick-borne virus in the presence of multiple host species: tick reservoirs and parasite mediated competition. J Theor Biol 200: 111–118. 10479543 10.1006/jtbi.1999.0982

[38] Begon M (2008) Effects of host diversity on disease dynamics. In:Ostfeld R S, Keesing F, Eviner V, (Eds), Infectious Disease Ecology: Effects of Ecosystems on Disease and of Disease on Ecosystems Princeton University Press, Princeton: 12-29.

[39] Hubalek Z, Sixl W, Halouzka J, Mikulaskova M (1997) Prevalence of Francisella tularensis in Dermacentor reticulatus ticks collected in adjacent areas of the Czech and Austrian Republics. Cent Eur J Public Health 5: 199–201. 9457420

[40] Grzeszczuk A (2006) Anaplasma phagocytophilum in Ixodes ricinus ticks and human granulocytic anaplasmosis seroprevalence among forestry rangers in Bialystok region. Adv Med Sci 51: 283–286. 17357327

[41] Moniuszko A, Zajkowska J, Pancewicz S, Kondrusik M, Grygorczuk S, et al (2011) Arthropod-borne tularemia in Poland: a case report. Vector Borne Zoonotic Dis 11: 1399–1401. 21612529 10.1089/vbz.2010.0227

[42] Chmielewska-Badora J, Moniuszko A, Zukiewicz-Sobczak W, Zwolinski J, Piatek J, et al (2012) Serological survey in persons occupationally exposed to tick-borne pathogens in cases of co-infections with Borrelia burgdorferi, Anaplasma phagocytophilum, Bartonella spp. and Babesia microti. Ann Agric Environ Med 19: 271–274. 22742800

[43] Ignatovich VF, Lukin EP, Umnova NS, Penkina GA, Vorob'ev AA (2001) [Seroimmunologic monitoring of microorganisms of Rickettsia and Bartonella species microorganisms in the Moscow region]. Zh Mikrobiol Epidemiol Immunobiol: 14-17. 11236493

[44] Foley JE, Nieto NC (2010) Tularemia. Vet Microbiol 140: 332–338. 19713053 10.1016/j.vetmic.2009.07.017

[45] Angelakis E, Raoult D (2010) Q Fever. Vet Microbiol 140: 297–309. 19875249 10.1016/j.vetmic.2009.07.016

[46] Alberti A, Zobba R, Chessa B, Addis MF, Sparagano O, et al (2005) Equine and canine Anaplasma phagocytophilum strains isolated on the island of Sardinia (Italy) are phylogenetically related to pathogenic strains from the United States. Appl Environ Microbiol 71: 6418–6422. 16204571 10.1128/AEM.71.10.6418-6422.2005 PMC1265917

[47] Casati S, Sager H, Gern L, Piffaretti JC (2006) Presence of potentially pathogenic Babesia sp. for human in Ixodes ricinus in Switzerland. Ann Agric Environ Med 13: 65–70. 16841874

[48] Zeaiter Z, Fournier PE, Raoult D (2002) Genomic variation of Bartonella henselae strains detected in lymph nodes of patients with cat scratch disease. J Clin Microbiol 40: 1023–1030. 11880432 10.1128/JCM.40.3.1023-1030.2002 PMC120271

[49] Clark K, Hendricks A, Burge D (2005) Molecular identification and analysis of Borrelia burgdorferi sensu lato in lizards in the southeastern United States. Appl Environ Microbiol 71: 2616–2625. 15870353 10.1128/AEM.71.5.2616-2625.2005 PMC1087528

[50] To H, Kako N, Zhang GQ, Otsuka H, Ogawa M, et al (1996) Q fever pneumonia in children in Japan. J Clin Microbiol 34: 647–651. 8904431 10.1128/jcm.34.3.647-651.1996 PMC228863

[51] Barns SM, Grow CC, Okinaka RT, Keim P, Kuske CR (2005) Detection of diverse new Francisella-like bacteria in environmental samples. Appl Environ Microbiol 71: 5494–5500. 16151142 10.1128/AEM.71.9.5494-5500.2005 PMC1214603

[52] Ishikura M, Ando S, Shinagawa Y, Matsuura K, Hasegawa S, et al (2003) Phylogenetic analysis of spotted fever group rickettsiae based on gltA, 17-kDa, and rOmpA genes amplified by nested PCR from ticks in Japan. Microbiol Immunol 47: 823–832. 14638993 10.1111/j.1348-0421.2003.tb03448.x

[53] Regnery RL, Spruill CL, Plikaytis BD (1991) Genotypic identification of rickettsiae and estimation of intraspecies sequence divergence for portions of two rickettsial genes. J Bacteriol 173: 1576–1589. 1671856 10.1128/jb.173.5.1576-1589.1991 PMC207306

[54] Roux V, Fournier PE, Raoult D (1996) Differentiation of spotted fever group rickettsiae by sequencing and analysis of restriction fragment length polymorphism of PCR-amplified DNA of the gene encoding the protein rOmpA. J Clin Microbiol 34: 2058–2065. 8862558 10.1128/jcm.34.9.2058-2065.1996 PMC229190

